# Supplementary material for: Influenza A and D Viruses in Non-Human Mammalian Hosts in Africa: A Systematic Review and Meta-Analysis
Source: Viruses. 2021 Dec 2;13(12):2411. doi: 10.3390/v13122411 (PMC8706448; doi:10.3390/v13122411)
Supplement: Supplementary file 1 [file viruses-13-02411-s001.zip › Manuscript Supplementary Materials.pdf]

Manuscript Supplementary Materials

Protocol S1: Detailed Literature Search Strategy

A systematic literature search was conducted to identify all publications reporting IAV and IDV in non-human mammalian species in Africa from the year 2000-2020. We searched literature from 2000-2020 because these are the two decades in which African countries reported a rise in influenza virus outbreaks in poultry and encompasses a period in which 2009 influenza pandemic (A(H1N1)pdm09) emerged." The following 13 search terms: "animal influenza viruses, influenza A virus, influenza D virus, influenza viruses, influenza A virus in animals, influenza D virus in animals, influenza in animals, influenza in swine, influenza A virus in pigs, influenza A virus in livestock, prevalence of influenza A virus in livestock, influenza outbreaks in livestock, epidemiology of influenza A virus in livestock and Africa". The Boolean terms "AND", "OR" and "NOT" were also used. Additional articles were identified by reviewing the reference list of the primary articles.

Checklist S2: Quality Assessment Checklist

The following items were examined and given a score based on a simple scale system (1 for "yes", 0 for "no").

1. Was the research objective clearly stated?
2. Was the sampling area clearly described with reference to the location, climate and level of development (rural, peri-urban or urban)?
3. Was the period of the study stated?
4. Was the target sample a close representation of the general population?
5. Was some form of random selection used to select the samples?
6. Was a minimum sample size calculated?
7. Were the sample processing and diagnostic method clearly described?
8. Were the subjects categorised by sex?
9. Were the subjects categorised by ownership and movement restriction and were the categories clearly defined?
10. Were the subjects categorised by age and were the age categories clearly defined?

The quality index score for each study was calculated by dividing the study quality score by 10.

Table S1: Studies included in the systematic review and meta-analysis

No.	Title of Study	Studies Included Meta-analysis (✓)
1	Abd El-Rahim, I. H. & Hussein, M. 2004. An epizootic of equine influenza in Upper Egypt in 2000. *Rev Sci Tech,* 23**,** 921-30 DOI: 10.20506/rst.23.3.1539.	
2	Abdel-Moneim, A. S., Abdel-Ghany, A. E. & Shany, S. A. 2010. Isolation and characterization of highly pathogenic avian influenza virus subtype H5N1 from donkeys. *J Biomed Sci,* 17**,** 25 DOI: 10.1186/1423-0127-17-25.	
3	Adeola, O.A.; Adeniji, J.A.; Olugasa, B.O. Isolation of influenza A viruses from pigs in Ibadan, Nigeria. *Vet Ital*. **2009**, *45*, 383-390.	✓
4	Adeola, O. A., Adeniji, J. A. & Olugasa, B. O. 2010. Detection of haemagglutination-inhibiting antibodies against human H1 and H3 strains of influenza A viruses in pigs in Ibadan, Nigeria. *Zoonoses Public Health,* 57**,** e89-94 DOI: 10.1111/j.1863-2378.2009.01268.x.	✓
5	Adeola, O. A., Olugasa, B. O. & Emikpe, B. O. 2016. Antigenic Detection of Human Strain of Influenza Virus A (H3N2) in Swine Populations at Three Locations in Nigeria and Ghana during the Dry Early Months of 2014. *Zoonoses Public Health,* 63**,** 106-11 DOI: 10.1111/zph.12210.	✓
6	Adeola, O. A., Olugasa, B. O. & Emikpe, B. O. 2017. Molecular detection of influenza A(H1N1)pdm09 viruses with M genes from human pandemic strains among Nigerian	✓

	pigs, 2013-2015: implications and associated risk factors. *Epidemiol Infect,* 145**,** 3345-3360 DOI: 10.1017/s0950268817002503.	
7	Aiki-Raji, C., Oyedele, I., Ayoade, G., Fagbohun, O. & Oderinu, T. 2004. Detection Of Haemagglutination–Inhibition Antibodies Against Human H 1 n 1 Strains Of Influenza A Viruses In Swine In Ibadan, Nigeria. *African Journal of Clinical and Experimental Microbiology,* 5**,** 278-279 DOI.	✓
8	Anjorin, A., Omilabu, S., Salu, O. & Oke, B. 2012. Detection of Influenza A Virus in Pigs in Lagos, Nigeria. *African Journal of Clinical and Experimental Microbiology,* 13**,** 41-45 DOI.	✓
9	Awosanya, E. J., Ogundipe, G., Babalobi, O. & Omilabu, S. 2013. Prevalence and correlates of influenza-A in piggery workers and pigs in two communities in Lagos, Nigeria. *Pan Afr Med J,* 16**,** 102 DOI: 10.11604/pamj.2013.16.102.1450.	✓
10	Ayim-Akonor, M., Mertens, E., May, J. & Harder, T. 2020. Exposure of domestic swine to influenza A viruses in Ghana suggests unidirectional, reverse zoonotic transmission at the human-animal interface. *Zoonoses Public Health,* 67**,** 697-707 DOI: 10.1111/zph.12751.	✓
11	Boukharta, M., Elharrak, M. & Ennaji, M. M. 2012. Seroepidemiological Study on Equine Influenza in Morocco-A/equin-1/Prague/56-A/equin-2/Miami/63 Seroepidemiological Study on Equine Influenza in Morocco *European Journal of Scientific Research,* 68**,** 147-153 DOI.	
12	Bunuma, E. K., Ochola, L. & Nyerere, A. K. 2018. A survey of influenza subtypes in olive baboons in selected areas in Kenya. *bioRxiv*, 380345 DOI.	
13	Cardinale, E., Pascalis, H., Temmam, S., Hervé, S., Saulnier, A., Turpin, M., Barbier, N., Hoarau, J., Quéguiner, S. & Gorin, S. 2012. Influenza a (H1N1) pdm09 virus in pigs, Reunion Island. *Emerging infectious diseases,* 18**,** 1665 DOI, https://www.ncbi.nlm.nih.gov/pmc/articles/PMC3471629/pdf/12-0398.pdf.	✓
14	Couacy-Hymann, E., Kouakou, V. A., Aplogan, G. L., Awoume, F., Kouakou, C. K., Kakpo, L., Sharp, B. R., Mcclenaghan, L., Mckenzie, P., Webster, R. G., Webby, R. J. & Ducatez, M. F. 2012. Surveillance for influenza viruses in poultry and swine, west Africa, 2006-2008. *Emerg Infect Dis,* 18**,** 1446-52 DOI: 10.3201/eid1809.111296.	✓
15	Daodu, O. B., Adebiyi, A. I. & Oluwayelu, D. O. 2019. Serological and molecular surveillance for influenza A virus in dogs and their human contacts in Oyo State, Nigeria. *Trop Biomed,* 36**,** 1054-1060 DOI.	
16	Dennis, K., Oyiguh, A. & Dadah, A. 2019. Seroprevalence Of Swine Influenza A Virus Circulating In Pigs From Southern Kaduna, Nigeria. *Science World Journal,* 14**,** 92-95 DOI.	✓
17	Diallo, A. A., Souley, M. M., Issa Ibrahim, A., Alassane, A., Issa, R., Gagara, H., Yaou, B., Issiakou, A., Diop, M., Ba Diouf, R. O., Lo, F. T., Lo, M. M., Bakhoum, T., Sylla, M., Seck, M. T., Meseko, C., Shittu, I., Cullinane, A., Settypalli, T. B. K., Lamien, C. E., Dundon, W. G. & Cattoli, G. 2020. Transboundary spread of equine influenza viruses (H3N8) in West and Central Africa: Molecular characterization of identified viruses during outbreaks in Niger and Senegal, in 2019. *Transbound Emerg Dis,* 10.1111/tbed.13779 DOI: 10.1111/tbed.13779.	
18	Dione, M., Masembe, C., Akol, J., Amia, W., Kungu, J., Lee, H. S. & Wieland, B. 2018. The importance of on-farm biosecurity: Sero-prevalence and risk factors of bacterial and viral pathogens in smallholder pig systems in Uganda. *Acta Trop,* 187**,** 214-221 DOI: 10.1016/j.actatropica.2018.06.025.	✓
19	Ducatez, M. F., Awoume, F. & Webby, R. J. 2015. Influenza A(H1N1)pdm09 virus in pigs, Togo, 2013. *Vet Microbiol,* 177**,** 201-5 DOI: 10.1016/j.vetmic.2015.02.028.	✓
20	El-Sayed, A., Awad, W., Fayed, A., Hamann, H. P. & Zschöck, M. 2010. Avian influenza prevalence in pigs, Egypt. *Emerg Infect Dis,* 16**,** 726-7 DOI: 10.3201/eid1604.091316.	✓
21	El-Sayed, A., Prince, A., Fawzy, A., Nadra, E., Abdou, M. I., Omar, L., Fayed, A. & Salem, M. 2013. Sero-prevalence of avian influenza in animals and human in Egypt. *Pak J Biol Sci,* 16**,** 524-9 DOI: 10.3923/pjbs.2013.524.529.	✓

22	Freidl, G. S., Binger, T., Müller, M. A., De Bruin, E., Van Beek, J., Corman, V. M., Rasche, A., Drexler, J. F., Sylverken, A., Oppong, S. K., Adu-Sarkodie, Y., Tschapka, M., Cottontail, V. M., Drosten, C. & Koopmans, M. 2015. Serological evidence of influenza A viruses in frugivorous bats from Africa. *PLoS One,* 10**,** e0127035 DOI: 10.1371/journal.pone.0127035.	
23	Fusade-Boyer, M.; Pato, P.S.; Komlan, M.; Dogno, K.; Batawui, K.; Go-Maro, E., *et al.* Risk Mapping of Influenza D Virus Occurrence in Ruminants and Swine in Togo Using a Spatial Multicriteria Decision Analysis Approach. *Viruses*. **2020**, *12*, DOI: 10.3390/v12020128.	✓
24	Gomaa, M. R., Kandeil, A., El-Shesheny, R., Shehata, M. M., Mckenzie, P. P., Webby, R. J., Ali, M. A. & Kayali, G. 2018. Evidence of infection with avian, human, and swine influenza viruses in pigs in Cairo, Egypt. *Arch Virol,* 163**,** 359-364 DOI: 10.1007/s00705-017-3619-3.	✓
25	Kandeil, A., Gomaa, M. R., Shehata, M. M., El Taweel, A. N., Mahmoud, S. H., Bagato, O., Moatasim, Y., Kutkat, O., Kayed, A. S., Dawson, P., Qiu, X., Bahl, J., Webby, R. J., Karesh, W. B., Kayali, G. & Ali, M. A. 2019. Isolation and Characterization of a Distinct Influenza A Virus from Egyptian Bats. *J Virol,* 93 DOI: 10.1128/jvi.01059-18.	
26	Kimber, K. R., Lubroth, J., Dubovi, E. J., Berninger, M. L. & Demaar, T. W. 2002. Serologic Survey of Selected Viral, Bacterial, and Protozoal Agents in Captive and Free-Ranging Ungulates from Central Kenya. *Annals of the New York Academy of Sciences,* 969**,** 217-223 DOI: https://doi.org/10.1111/j.1749-6632.2002.tb04382.x.	
27	Kirunda, H., Erima, B., Tumushabe, A., Kiconco, J., Tugume, T., Mulei, S., Mimbe, D., Mworozi, E., Bwogi, J., Luswa, L., Kibuuka, H., Millard, M., Byaruhanga, A., Ducatez, M. F., Krauss, S., Webby, R. J., Webster, R. G., Wurapa, K., Byarugaba, D. K. & Wabwire-Mangen, F. 2014. Prevalence of influenza A viruses in livestock and free-living waterfowl in Uganda. *BMC Vet Res,* 10**,** 50 DOI: 10.1186/1746-6148-10-50.	✓
28	Laing, G., Christley, R., Stringer, A., Aklilu, N., Ashine, T., Newton, R., Radford, A. & Pinchbeck, G. 2018. Respiratory disease and sero-epidemiology of respiratory pathogens in the working horses of Ethiopia. *Equine Vet J,* 50**,** 793-799 DOI: 10.1111/evj.12834.	
29	Larison, B., Njabo, K. Y., Chasar, A., Fuller, T., Harrigan, R. J. & Smith, T. B. 2014. Spillover of pH1N1 to swine in Cameroon: an investigation of risk factors. *BMC Vet Res,* 10**,** 55 DOI: 10.1186/1746-6148-10-55.	
30	Meseko, C., Cilloni, F. & Oladokun, A. 2012. Serosurvey of antibody to highly pathogenic avian influenza (H5N1) in pigs, north central Nigeria. *Sokoto Journal of Veterinary Sciences,* 10**,** 52-55 DOI.	✓
31	Meseko, C., Globig, A., Ijomanta, J., Joannis, T., Nwosuh, C., Shamaki, D., Harder, T., Hoffman, D., Pohlmann, A., Beer, M., Mettenleiter, T. & Starick, E. 2018. Evidence of exposure of domestic pigs to Highly Pathogenic Avian Influenza H5N1 in Nigeria. *Sci Rep,* 8**,** 5900 DOI: 10.1038/s41598-018-24371-6.	✓
32	Meseko, C. A., Ehizibolo, D. O., Nwokike, E. C. & Wungak, Y. S. 2016. Serological evidence of equine influenza virus in horse stables in Kaduna, Nigeria. *J Equine Sci,* 27**,** 99-105 DOI: 10.1294/jes.27.99.	
33	Meseko, C. A., Odaibo, G. N. & Olaleye, D. O. 2014. Detection and isolation of 2009 pandemic influenza A/H1N1 virus in commercial piggery, Lagos Nigeria. *Vet Microbiol,* 168**,** 197-201 DOI: 10.1016/j.vetmic.2013.11.003.	✓
34	Munyua, P., Onyango, C., Mwasi, L., Waiboci, L. W., Arunga, G., Fields, B., Mott, J. A., Cardona, C. J., Kitala, P., Nyaga, P. N. & Njenga, M. K. 2018. Identification and characterization of influenza A viruses in selected domestic animals in Kenya, 2010-2012. *PLoS One,* 13**,** e0192721 DOI: 10.1371/journal.pone.0192721.	✓
35	Murakami, S.; Endoh, M.; Kobayashi, T.; Takenaka-Uema, A.; Chambers, J.K.; Uchida, K., *et al.* Influenza D Virus Infection in Herd of Cattle, Japan. *Emerg Infect Dis*. **2016**, *22*, 1517-1519, DOI: 10.3201/eid2208.160362.	✓

36	Njabo, K. Y., Fuller, T. L., Chasar, A., Pollinger, J. P., Cattoli, G., Terregino, C., Monne, I., Reynes, J. M., Njouom, R. & Smith, T. B. 2012. Pandemic A/H1N1/2009 influenza virus in swine, Cameroon, 2010. *Vet Microbiol,* 156**,** 189-92 DOI: 10.1016/j.vetmic.2011.09.003.	✓
37	Oluwayelu, D. O., Bankole, O., Ajagbe, O., Adebiyi, A. I., Abiola, J. O., Otuh, P. & Omobowale, O. T. 2014. Serological survey for emerging canine H3N8 and H3N2 influenza viruses in pet and village dogs in Nigeria. *Afr J Med Med Sci,* 43 Suppl**,** 111-5 DOI.	
38	Osoro, E. M., Lidechi, S., Marwanga, D., Nyaundi, J., Mwatondo, A., Muturi, M., Ng'ang'a, Z. & Njenga, K. 2019. Seroprevalence of influenza A virus in pigs and low risk of acute respiratory illness among pig workers in Kenya. *Environ Health Prev Med,* 24**,** 53 DOI: 10.1186/s12199-019-0808-6.	✓
39	Osoro, E. M., Lidechi, S., Nyaundi, J., Marwanga, D., Mwatondo, A., Muturi, M., Ng'ang'a, Z. & Njenga, K. 2019. Detection of pandemic influenza A/H1N1/pdm09 virus among pigs but not in humans in slaughterhouses in Kenya, 2013-2014. *BMC Res Notes,* 12**,** 628 DOI: 10.1186/s13104-019-4667-4.	✓
40	Saegerman, C., Salem, E., Ait Lbacha, H., Alali, S., Zouagui, Z., Meyer, G. & Ducatez, M. F. 2020. Formal estimation of the seropositivity cut-off of the hemagglutination inhibition assay in field diagnosis of influenza D virus in cattle and estimation of the associated true prevalence in Morocco. *Transbound Emerg Dis,* 10.1111/tbed.13805 DOI: 10.1111/tbed.13805.	✓
41	Salem, E.; Cook, E.A.J.; Lbacha, H.A.; Oliva, J.; Awoume, F.; Aplogan, G.L., *et al.* Serologic Evidence for Influenza C and D Virus among Ruminants and Camelids, Africa, 1991-2015. *Emerg Infect Dis*. **2017**, *23*, 1556-1559, DOI: 10.3201/eid2309.170342.	✓
42	Shittu, I., Meseko, C. A., Sulaiman, L. P., Inuwa, B., Mustapha, M., Zakariya, P. S., Muhammad, A. A., Muhammad, U., Atuman, Y. J., Barde, I. J., Zecchin, B., Quaranta, E. G., Shamaki, D., Alabi, O., Monne, I., Fusaro, A. & Joannis, T. M. 2020. Fatal multiple outbreaks of equine influenza H3N8 in Nigeria, 2019: The first introduction of Florida clade 1 to West Africa. *Vet Microbiol,* 248**,** 108820 DOI: 10.1016/j.vetmic.2020.108820.	
43	Snoeck, C. J., Abiola, O. J., Sausy, A., Okwen, M. P., Olubayo, A. G., Owoade, A. A. & Muller, C. P. 2015. Serological evidence of pandemic (H1N1) 2009 virus in pigs, West and Central Africa. *Vet Microbiol,* 176**,** 165-71 DOI: 10.1016/j.vetmic.2014.12.022.	
44	Soilemetzidou, E. S., De Bruin, E., Franz, M., Aschenborn, O. H. K., Rimmelzwaan, G. F., Van Beek, R., Koopmans, M., Greenwood, A. D. & Czirják, G. 2020. Diet May Drive Influenza A Virus Exposure in African Mammals. *J Infect Dis,* 221**,** 175-182 DOI: 10.1093/infdis/jiz032.	
45	Tialla, D., Sausy, A., Cissé, A., Sagna, T., Ilboudo, A. K., Ouédraogo, G. A., Hübschen, J. M., Tarnagda, Z. & Snoeck, C. J. 2020. Serological evidence of swine exposure to pandemic H1N1/2009 influenza A virus in Burkina Faso. *Vet Microbiol,* 241**,** 108572 DOI: 10.1016/j.vetmic.2019.108572.	✓
46	Equine Influenza Virus in Tunisia. Immediate Notification, Report ID: IN_6088, Report Date: 16/06/2005 [Internet]. World Organisation for Animal Health (OIE). 2005 [cited 23 November 2021]. Available from: https://wahis.oie.int/#/report-info?reportId=21460.	
47	Equine Influenza Virus in Egypt. Follow-up report 3 (Final report), Report ID: FUR_7406, Report Date: 07/10/2008 [Internet]. World Organisation for Animal Health. 2008 [cited 23 November 2021]. Available from: https://wahis.oie.int/#/report-info?reportId=1546.	
48	Equine Influenza Virus in Mali. Immediate notification (Final report), Report ID: IN_31526, Report date: 22/08/2019 [Internet]. World Organisation for Animal Health (OIE). 2019 [cited 23 November 2021]. Available from: https://wahis.oie.int/#/report-info?reportId=24966.	
49	Equine Influenza Virus in Sudan. Immediate notification (Final report), Report ID: IN_30986, Report Date: 05/07/2019 [Internet]. World Organisation for Animal Health (OIE). 2019 [cited 23 November 2021]. Available from: https://wahis.oie.int/#/report-info?reportId=22040.	

Table S2: Characteristics of the included studies

Characteristics	Studies n (%)
**Animal Species** [1]	
Pigs	28 (45.2)
Cattle	4 (6.5)
Small ruminants – Goat and Sheep	4 (6.5)
Camels	3 (4.8)
Equids (Donkeys, Horses, Mule)	12 (19.4)
Domestic Canine – Cats and Dogs	4 (6.5)
Rats	1 (1.6)
Olive baboons	1 (1.6)
Zebra	1 (1.6)
Buffaloes	1 (1.6)
Bats	2 (3.2)
Herbivores – African elephant, Springbok, Black Rhinos and Wildebeest	1 (1.6)
Carnivores – Black rhino, Brown hyena, Spotted hyena, Bat-eared fox, Lion, Leopard, Cheetah, Caracal and Black-backed Jackal	1 (1.6)
**Type of Samples**	
Nasal Swabs	11 (22.4)
Serum	17 (34.7)
Mixed [2]	18 (36.7)
Unknown	3 (6.1)
**Method of Testing**	
Haemagglutinin Inhibition (HI)	7 (14.3)
ELISA	11 (22.4)
Virus Neutralisation Assay	1 (2.0)
Protein Microarray	1 (2.0)
RT-PCR	6 (11.1)
Culture	1 (2.0)
Mixed [3]	22 (44.9)
**Influenza Viruses**	
Influenza A virus	41 (83.7)
Influenza D virus	4 (8.2)
ND [4]	4 (8.2)
**Healthy Status**	
Asymptomatic (Apparently healthy)	14 (28.6)
Symptomatic	9 (18.4)
Mixed [5]	2 (4.1)
Not reported	24 (48.9)
**Vaccination Status**	
Vaccinated	1 (2.0)
Not Vaccinated	14 (28.6)
Not Reported	34 (69.4)
**Types of Study**	
Cross-sectional	23 (46.9)
Longitudinal	2 (4.1)
Surveillance	20 (40.8)
Reports	4 (8.2)

[1] Some studies reported more than one animal species, [2] Mixed means two types of samples were collected i.e., nasal swab and serum, [3] Two or more methods were used, [4] ND – Not Detected, [5] Asymptomatic and symptomatic population.

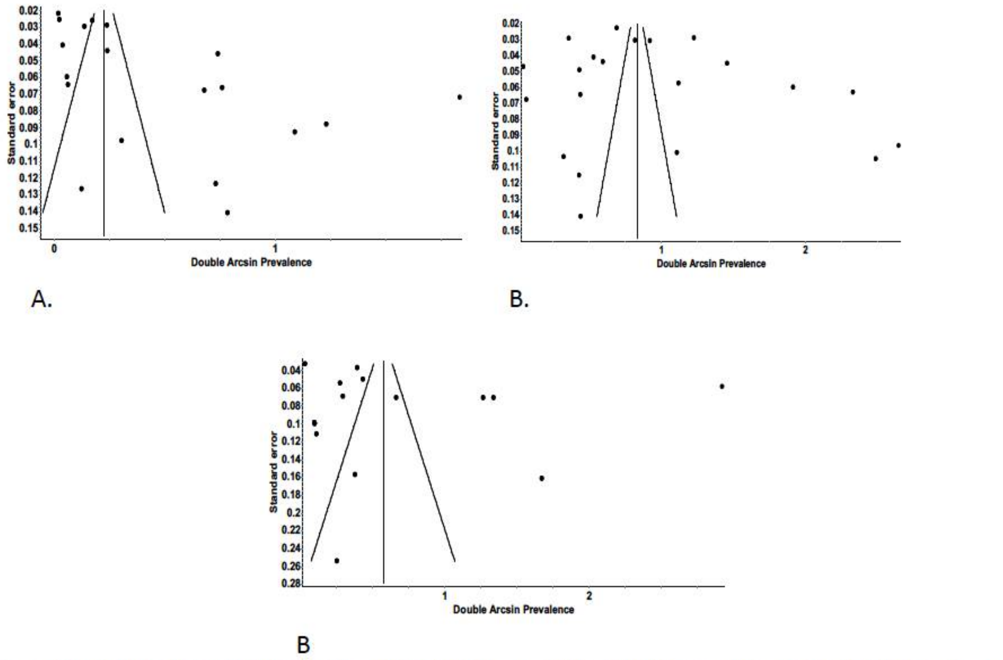

Figure S1: Funnel plots used to examine publication bias. A: Publication bias in studies related to IAV in pigs in Africa; B: Publication bias in studies related to seroprevalence of IAV in pigs in Africa; C: Publication bias related to seroprevalence of IDV in non-avian species in Africa.

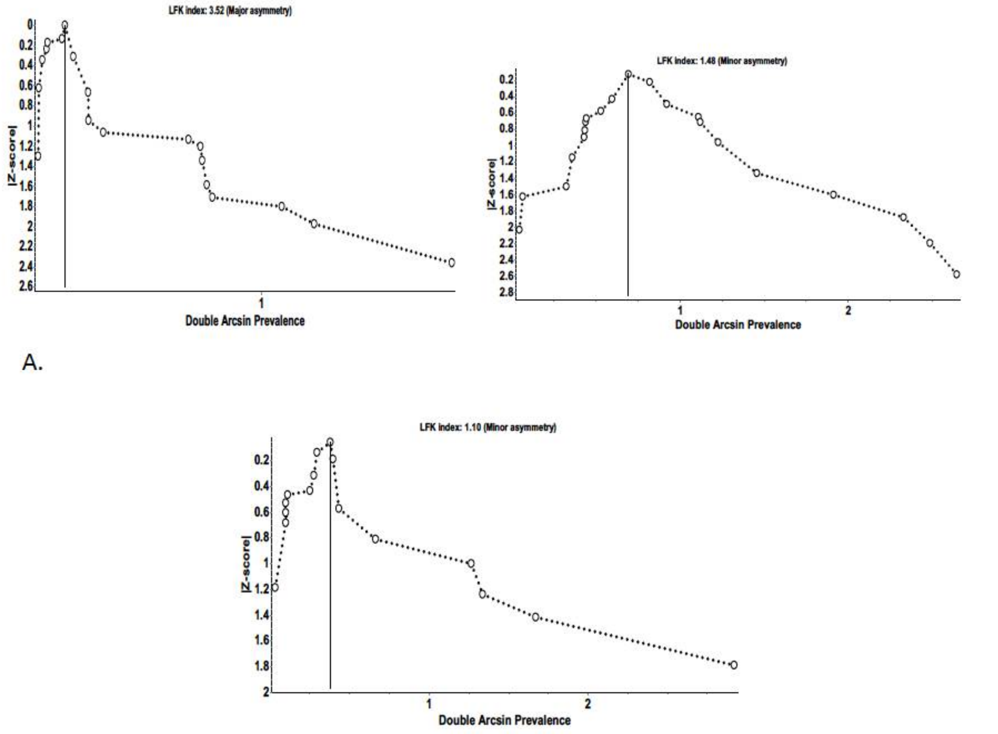

Figure S2: Doi plots with pseudo 95% confidence limits used to examine publication bias. A: Publication bias in studies related to IAV in pigs in Africa; B: Publication bias in studies related to seroprevalence of IAV in pigs in Africa; C: Publication bias related to seroprevalence of IDV in non-avian species in Africa.
